# 3D endoscopy shows enhanced anatomical details and depth perception vs 2D: a multicentre study

**DOI:** 10.1007/s00405-020-06495-6

**Published:** 2020-12-29

**Authors:** Peter Valentin Tomazic, Fabian Sommer, Andreas Treccosti, Hans Rudolf Briner, Andreas Leunig

**Affiliations:** 1 Dept. of General Otorhinolaryngology, Medical University of Graz, Auenbruggerplatz 26, 8036 Graz, Austria; 2 Dept. of Otorhinolaryngology, Head and Neck Surgery, Ulm University Medical Center, Frauensteige 12, 89075 Ulm, Germany; 3 Center for Otorhinolaryngology, Head and Neck Surgery, Klinik Hirslanden, Witellikerstrasse 40, CH-8032 Zurich, Switzerland; 4 Rhinology Center Munich, Platzl 3, 80331 Munich, Germany

**Keywords:** Anatomy, Imaging, Feasibility, Functional endoscopic sinus surgery, Standard endoscopy

## Abstract

**Purpose:**

The current standard endoscopic technique is a high resolution visualisation up to Full HD and even 4 K. A recent development are 3D endoscopes providing a 3-dimensional picture, which supposedly gives additional information of depth, anatomical details and orientation in the surgical field. Since the 3D-endoscopic technique is new, little scientific evidence is known whether the new technique provides advantages for the surgeon compared to the 2D-endoscopic standard technique in FESS. This study compares the standard 2D-endoscopic surgical technique with the new commercially available 3D-endoscopic technique.

**Methods:**

The prospective randomized interventional multicenter study included a total of 80 referred patients with chronic rhinosinusitis with and without polyps without prior surgery. A bilateral FESS procedure was performed, one side with the 2D-endoscopic technique, the other side with the 3D-endoscopic technique. The time of duration was measured. Additionally, a questionnaire containing 20 items was completed by 4 different surgeons judging subjective impression of visualisation and handling.

**Results:**

2D imaging was superior to 3D apart from “recognition of details”, “depth perception” and “3D effect”. For usability properties 2D was superior to 3D apart from “weight of endoscopes”. Mean duration for surgery was 26.1 min for 2D and 27.4 min. for 3D without statistical significance (*P* = 0.219).

**Conclusion:**

Three-dimensional endoscopy features improved depth perception and recognition of anatomic details but worse overall picture quality. It is useful for teaching purposes, yet 2D techniques provide a better outcome in terms of feasibility for routine endoscopic approaches.

**Electronic supplementary material:**

The online version of this article (10.1007/s00405-020-06495-6) contains supplementary material, which is available to authorized users.

## Introduction

Chronic rhinosinusitis is a common disease with a prevalence of 3–5% and may lead to a significant impairment of the quality of life in individuals suffering from the disorder. The disease is clinically defined by following symptoms: blocked/congested nose, rhinorrhea (anterior or posterior nasal drip) as well as hyposmia, facial pain or pressure or cough in children for more than 12 weeks where either congestions or rhinorrhea have to be present [1]. Medical therapy with topical steroids and nasal rinses are the main treatment of choice to reduce the symptoms. In severe disease, however, medical therapy may not be sufficient to control the disease activity and symptoms. In these cases, surgical therapy is indicated. The principle of surgical therapy is to open the narrow and blocked drainage pathways of the paranasal sinuses and thereby restoring mucociliary clearance and widening the access for topical medical treatment of the diseased mucosa in the paranasal sinuses. This procedure is known as “Functional Endoscopic Sinus Surgery” (FESS) and it is generally accepted and recommended by international guidelines as procedure of choice in chronic rhinosinusitis not responding sufficiently to medical treatment [2, 3]. Modern visualisation and navigation technologies (help) support to reduce the potential risks of FESS such as injury of the orbit, the optic nerve, the carotid artery, the skull base and the brain which are in close topographical relationship to the paranasal sinuses [1, 4]. A key component in endoscopic sinus surgery is the endoscope and camera which enables a good visualisation of the surgical field. The current standard technique of endoscopic visualisation is using endoscopes combined with 4 K resolution camera, providing a 2-dimensional (2D) picture on a high resolution (4 K) screen. A recent development are 3D endoscopes providing a 3-dimensional picture, which supposedly gives additional information of depth, anatomical details and orientation in the surgical field. This “3D”—technology consists of special two lens digital endoscopes combined with a 3D camera and the surgeon wears glasses to enable a 3D visualisation on screen. The visualisation of a 3-dimensional surgical field has the theoretical advantage to provide the surgeon with more realistic information about the anatomy of the surgical field which may be beneficial for surgical control and may even reduce complications.

Since the 3D-endoscopic technique is new, little scientific evidence is known whether the new technique provides advantages for the surgeon compared to the 2D-endoscopic standard technique in FESS.

This study compares the standard 2D-endoscopic surgical technique with the new commercially available 3D-endoscopic technique.

## Materials and methods

The study was designed as open prospective randomized interventional study in an international multicenter setting.

**Fig.1:**
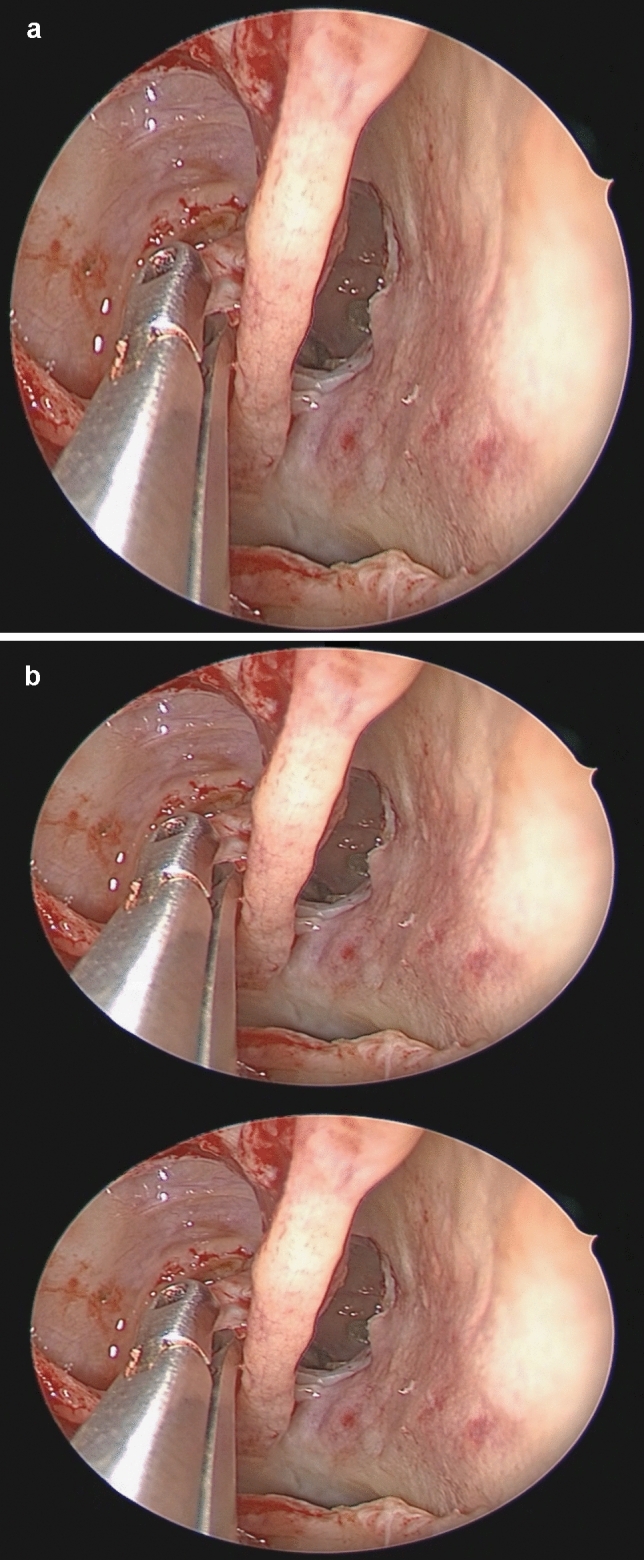
**a** 2D image of the a right posterior ethmoid with the superior turbinate and the enlarged sphenoid sinus drainage pathway, while removing small bone parts of the 4th lamella. **b** 3D image of the same localisation, by using 3D glasses and monitors the two images are superimposed to create a 3D effect

**Fig. 2:**
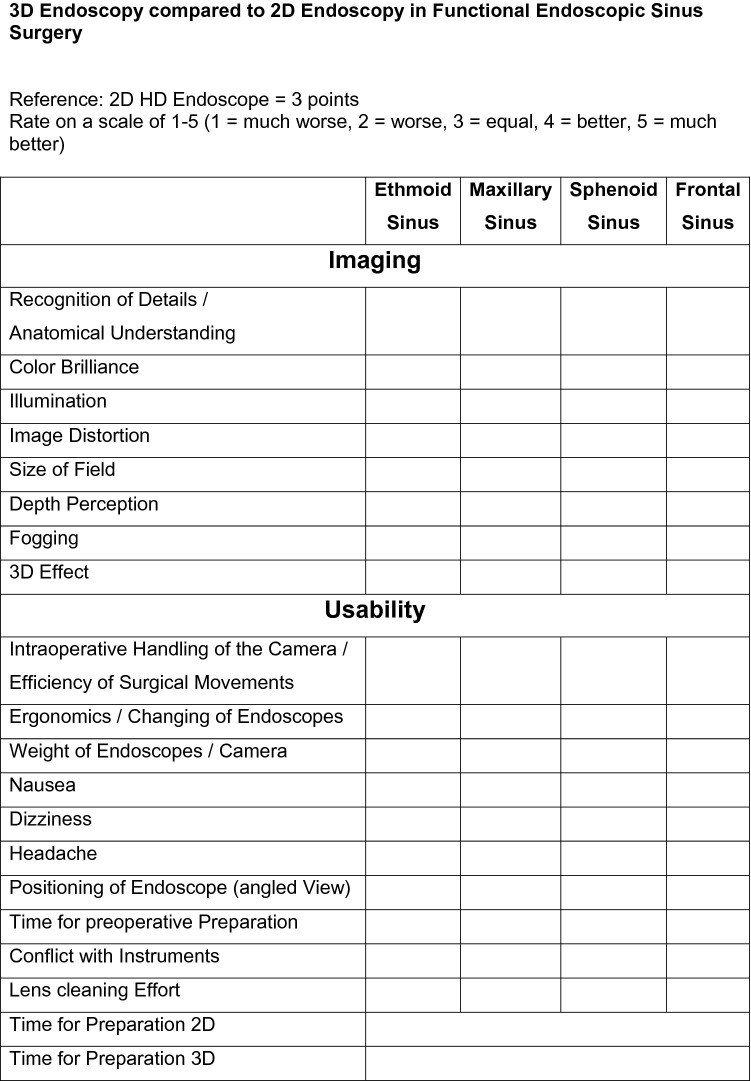
Questionnaire for the surgeon postoperatively with items to evaluate imaging and usability

A total of 80 referred patients (20 per centre) with chronic rhinosinusitis with and without polyps without prior surgery but refractory to conservative treatment were included according to EPOS2020 guidelines. Following exclusion criteria were applied: age below 18 years, previous sinus operations, unilateral or asymmetric disease, and severe comorbidities such as bleeding disorders and inability or unwillingness to give consent for the study. A bilateral FESS procedure was performed, one side with the 2D-endoscopic technique, the other side with the 3D-endoscopic technique which was randomized by means of an electronic randomization programme where IDs together with sides were entered in subsequent order and sides were then randomized to either 2D or 3D. The FESS procedure was performed on one side with the standard KARL STORZ 2D/HD endoscopic camera whereas the other side was operated with the new 3D endoscopic camera devices (TIPCAM^®^1 S 3D, 30°, 4 mm; TIPCAM^®^1 S 3D, 0°, 4 mm, Karl Storz GmbH) (Fig. 1a, b). The time of duration for the procedure using the 2D-endoscope and the 3D-endoscope was measured per side. A questionnaire was completed by the surgeon judging the subjective impression of visualisation and handling after surgery (Fig. 2). The questionnaire comprised 20 questions with an ordinal scale from 1–5 where 1 meant that 3D was much worse than 2D and 5 meant that 3D was much better than 2D. Values are presented as means with standard deviation. No further interventions or controls were performed and clinical outcome was not evaluated.

Four (PVT, FS, AL and HRB) rhinosurgeons at four centers (Graz/AT, Ulm/GER, Munich/GER, Zurich/SUI) operated and evaluated 20 patients each who were referred for FESS. Values are presented as means/medians with standard deviation (SD)/ranges and percentages where applicable. For statistical analysis between surgeons Mann–Whitney *U* tests (imaging and usability scores) or student *t* tests (duration of surgery) were applied. Institutional review board and ethical approval was obtained from all centres.

## Results

**Fig. 3:**
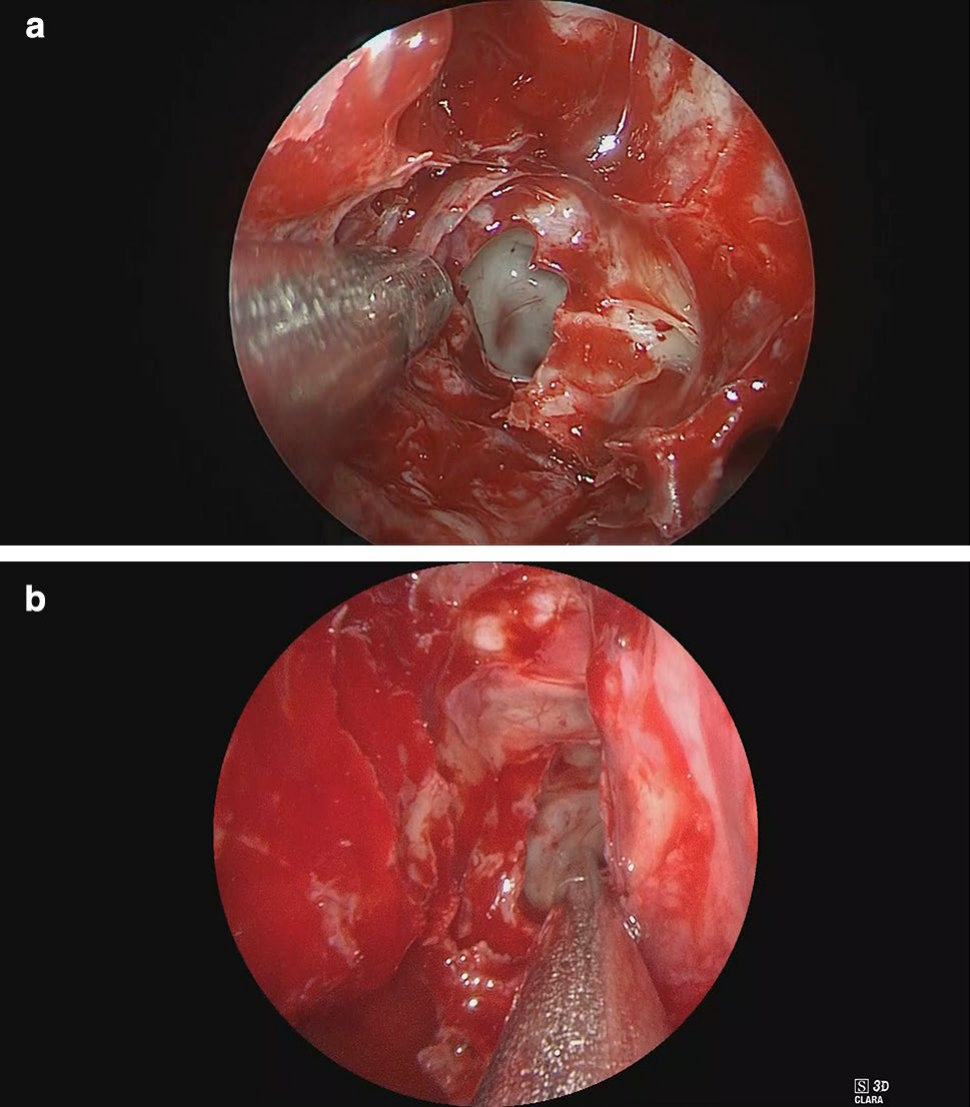
Example of 2D (**a**) where colour brilliance and brightness is superior to 3D (**b**)

**Fig. 4:**
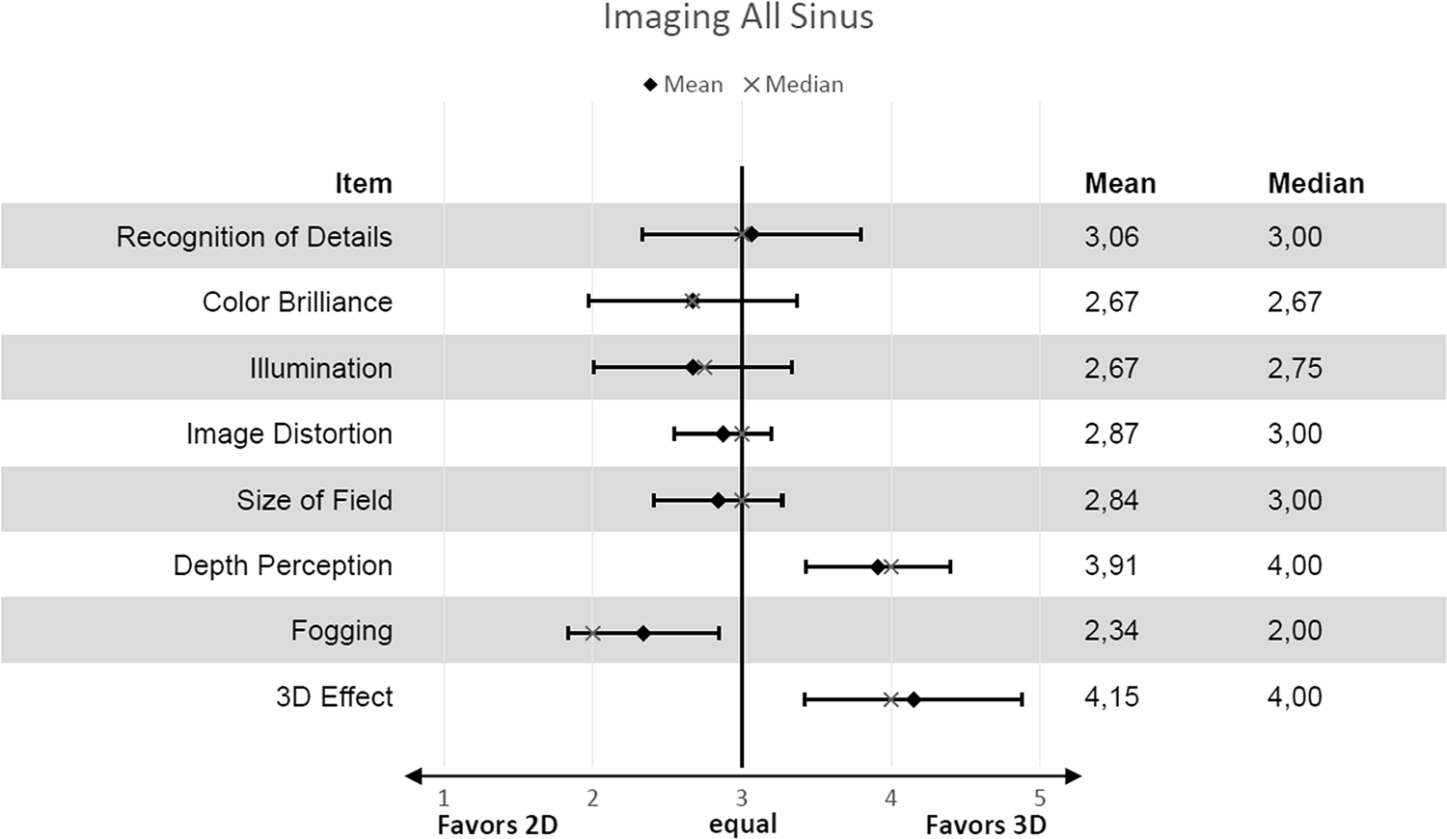
Results for imaging presented with means and standard deviation as well as medians from a 5 point scale. The graph is similar to a forest blot where 3 points on the *x*-axis mean 2D is equal to 3D. All items are evaluated on a 5 point Likert-like scale where 1 means 2D is highly superior to 3D and 5 3D is highly superior to 2D

**Fig. 5:**
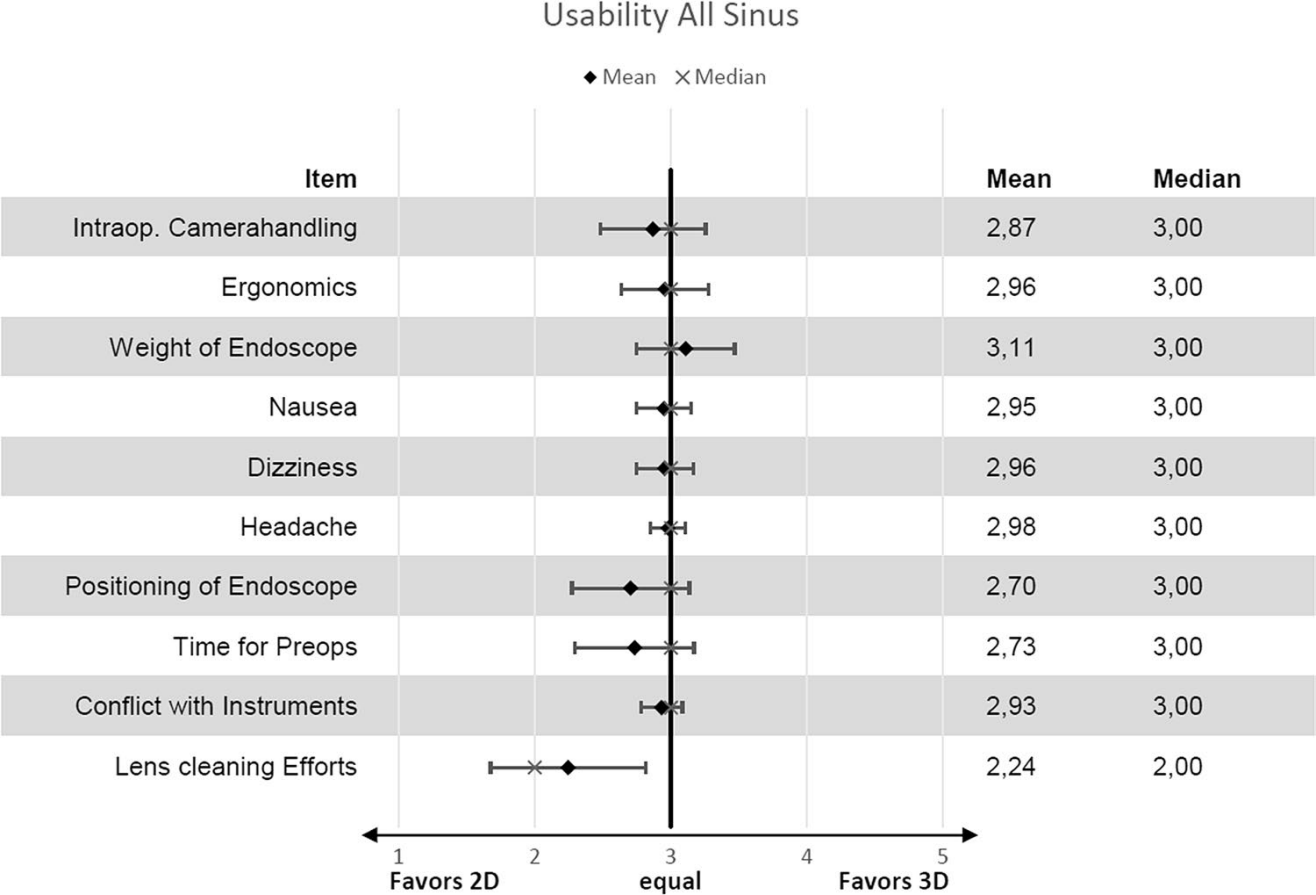
Results for usability presented with means and standard deviation as well as medians from a 5 point scale. The graph is similar to a forest blot where 3 points on the *x*-axis mean 2D is equal to 3D. All items are evaluated on a 5 point Likert-like scale where 1 means 2D is highly superior to 3D and 5 3D is highly superior to 2D

In this study, 80 patients were included and 70 ethmoid (anterior and posterior) and maxillary sinuses, 60 sphenoid sinuses and 61 frontal sinuses evaluated. For imaging properties (Figs. 3a, b) 2D was superior to 3D apart from “recognition of details” (mean 3.1; SD 0.73), “depth perception” (mean 3.9; SD 0.48) and “3D effect” (mean 4.1; SD 0.73) (Fig. 4). Detailed analysis of individual sinuses is shown in supplementary tables (Suppl. Table E1). For usability properties 2D was superior to 3D apart from “weight of endoscopes” (mean 3.1; SD 0.04) (Fig. 5). Detailed analysis of individual sinuses is shown in supplementary tables (Suppl. Table E2). Between surgeons there was no significant difference in scores (*P* = 0.187). Mean duration for surgery was 26.1 min (SD 12.37) for 2D and 27.4 min. (SD 15.45) for 3D without statistical significance (*P* = 0.219). The mean duration for surgery (per sinus) was 7.75 min (SD 4.38) for 2D and 8.06 min. (SD 5.76) for 3D without statistical significance (*P* = 0.334).

No major complications were reported.

## Discussion

The advent and further development of endoscopic techniques of the sinuses and later the anterior skull base came along with the debate about the superiority of endoscopic versus microscopic techniques [5, 6]. Despite the fact that endoscopic transnasal techniques have become the gold standard for approaches to the anterior skull base studies still compare both techniques with similar outcomes [7].

The most compelling argument for using the microscope was the 3-dimensional image of the surgical field. Recently, digital 3D endoscopes have been development to also overcome this shortcoming of the endoscopic technique. For skull base surgery particularly depth perception was a clear advantage of the novel 3D technique [8–10]. For sinus surgery itself, little is published about the feasibility of 3D techniques. In 2015, Ogino-Nishimura et al. [11] analysed various approaches in five cadavers and compared 2D to 3D techniques. They concluded that 3D offered a more precise anatomical understanding especially of the posterior paranasal sinus structures. To our knowledge, this is the first prospective multicentre study analysing the feasibility of 2D versus 3D FESS. We focused on imaging and handling aspects of both systems from KARL STORZ and answered questionnaires after each surgery. As expected, 3D showed a better score in 3D effect. As in other studies, recognition of details and depth perception was superior in 3D than in 2D. Only in the maxillary sinus recognition of details was slightly better in 2D, perhaps due to lack of protruding structures into the sinus lumen compared to other sinuses (e.g. optic nerve and carotid bulges in sphenoid) with flattened walls. The sphenoid sinuses showed the highest score (mean 4.11) in depth perception which is relevant for endoscopic sinus surgery as well as skull base surgery given the anatomic vicinity of both optic nerves and carotid arteries, the latter often with the inherited risk of serious bleeding [12]. We also analysed the results between all surgeons to rule out the possibility of bias in favour of 2D endoscopy, which was used for decades. All surgeons were instructed and trained with 3D technique to overcome the bias of long-term prior application of 2D as well as a potential learning curve which would have influenced the results over time. Incidentally, this is a similar argument that was originally raised when microscopes challenged with endoscopes. Since 2D scored better in all aspects but weight of the endoscopes concerning handling and user-friendliness we were interested in duration of surgery. Here, no significant differences could be seen comparing the two systems where 2D was slightly more time sparing. The time differences between the groups comparing time used per single sinuses were not as strong compared to all sinuses. This may be explained by the fact that some patients required more extensive surgery with an overall longer time.

The biggest advantage of 3D thus lies in anatomical details and depth perception. Here, especially for teaching purposes 3D is a useful tool to highlight the topographical relation to critical anatomical structures within the paranasal sinus system. A disadvantage is the handling especially the need to continuously wear 3D glasses, the extra effort in lens cleaning to the easier fogging of one of the lenses and the hitherto worsening of image quality and the changing of the scopes which need to be plugged in at the consoles whereas in 2D the surgeon can easily switch angled scopes himself. Another disadvantage is that the scope and camera are one single piece. Especially in angled endoscopy you either need to turn the endoscope for upward and sideward view which results in a rotation of the entire image or you digitally change the orientation of the scope.

The results of this study indicate that 3D endoscopic technology is a useful adjunct to standard techniques which may have a benefit for teaching purposes and anatomic understanding of the paranasal sinuses and skull base. However, a shortcoming of the present analysis is the lack of outcome data which were not evaluated in this feasibility study. Further prospective studies are needed to show if there is any superiority of 3D when it comes to complications or even surgical outcomes.

## Conclusion

Three-dimensional endoscopy shows an improved depth perception and recognition of anatomic details. It is useful for teaching purposes, yet 2D techniques show a better overall outcome in terms of feasibility for routine endoscopic approaches.

## Electronic supplementary material

Below is the link to the electronic supplementary material.

Supplementary file1 (DOCX 30 KB)

Supplementary file2 (DOCX 29 KB)
