# Supplementary material for: 3D endoscopy shows enhanced anatomical details and depth perception vs 2D: a multicentre study
Source: Eur Arch Otorhinolaryngol. 2020 Dec 29;278(7):2321–6. doi: 10.1007/s00405-020-06495-6 (PMC8165070; doi:10.1007/s00405-020-06495-6)
Supplement: Supplementary file 1 — Supplementary file1 (DOCX 30 KB) [file 405_2020_6495_MOESM1_ESM.docx]

| **Statistics Usability - Ethmoid Sinus** | | | | | | | | | | | |
| --- | --- | --- | --- | --- | --- | --- | --- | --- | --- | --- | --- |
|  | | ES Intraoperative Camerahandling | ES Ergonomics | ES Weight of Endoscope | ES Nausea | ES Dizziness | ES Headache | ES Positioning of Endoscope | ES Time for Preops | ES Conflict with Instruments | ES Lens cleaning Effort |
| N | Valid | 78 | 78 | 78 | 78 | 78 | 78 | 77 | 78 | 78 | 78 |
|  | Invalid | 2 | 2 | 2 | 2 | 2 | 2 | 3 | 2 | 2 | 2 |
| Mean | | 2,9231 | 2,9872 | 3,1026 | 2,9359 | 2,9615 | 2,9872 | 2,9091 | 2,7308 | 2,9615 | 2,2692 |
| Standarderror of the Mean | | ,05404 | ,03411 | ,04684 | ,02791 | ,02192 | ,01282 | ,04596 | ,05055 | ,02192 | ,06748 |
| Median | | 3,0000 | 3,0000 | 3,0000 | 3,0000 | 3,0000 | 3,0000 | 3,0000 | 3,0000 | 3,0000 | 2,0000 |
| Std.-Deviation | | ,47725 | ,30124 | ,41372 | ,24652 | ,19355 | ,11323 | ,40334 | ,44643 | ,19355 | ,59594 |
| Minimum | | 2,00 | 2,00 | 2,00 | 2,00 | 2,00 | 2,00 | 2,00 | 2,00 | 2,00 | 1,00 |
| Maximum | | 4,00 | 4,00 | 4,00 | 3,00 | 3,00 | 3,00 | 4,00 | 3,00 | 3,00 | 4,00 |
| Sum | | 228,00 | 233,00 | 242,00 | 229,00 | 231,00 | 233,00 | 224,00 | 213,00 | 231,00 | 177,00 |
| Percentile | 25 | 3,0000 | 3,0000 | 3,0000 | 3,0000 | 3,0000 | 3,0000 | 3,0000 | 2,0000 | 3,0000 | 2,0000 |
|  | 50 | 3,0000 | 3,0000 | 3,0000 | 3,0000 | 3,0000 | 3,0000 | 3,0000 | 3,0000 | 3,0000 | 2,0000 |
|  | 75 | 3,0000 | 3,0000 | 3,0000 | 3,0000 | 3,0000 | 3,0000 | 3,0000 | 3,0000 | 3,0000 | 3,0000 |

| **Statistics Usability – Maxillary Sinus** | | | | | | | | | | | |
| --- | --- | --- | --- | --- | --- | --- | --- | --- | --- | --- | --- |
|  | | MS Intraoperative Camerahandling | MS Ergonomics | MS Weight of Endoscope | MS Nausea | MS Dizziness | MS Headache | MS Positioning of Endoscope | MS Time for_Preops | MS Conflict with Instruments | MS Lens cleaning Effort |
| N | Valid | 78 | 78 | 78 | 78 | 78 | 78 | 77 | 78 | 78 | 78 |
|  | Invalid | 2 | 2 | 2 | 2 | 2 | 2 | 3 | 2 | 2 | 2 |
| Mean | | 2,8205 | 2,9359 | 3,1026 | 2,9615 | 2,9615 | 2,9872 | 2,4935 | 2,7051 | 2,9103 | 2,2436 |
| Standarderror of the Mean | | ,05078 | ,04595 | ,04314 | ,02192 | ,02192 | ,01282 | ,08189 | ,05196 | ,04156 | ,06370 |
| Median | | 3,0000 | 3,0000 | 3,0000 | 3,0000 | 3,0000 | 3,0000 | 3,0000 | 3,0000 | 3,0000 | 2,0000 |
| Std.-Deviation | | ,44848 | ,40579 | ,38104 | ,19355 | ,19355 | ,11323 | ,71861 | ,45894 | ,36701 | ,56260 |
| Minimum | | 2,00 | 2,00 | 2,00 | 2,00 | 2,00 | 2,00 | 1,00 | 2,00 | 1,00 | 1,00 |
| Maximum | | 4,00 | 4,00 | 4,00 | 3,00 | 3,00 | 3,00 | 3,00 | 3,00 | 3,00 | 4,00 |
| Sum | | 220,00 | 229,00 | 242,00 | 231,00 | 231,00 | 233,00 | 192,00 | 211,00 | 227,00 | 175,00 |
| Percentile | 25 | 3,0000 | 3,0000 | 3,0000 | 3,0000 | 3,0000 | 3,0000 | 2,0000 | 2,0000 | 3,0000 | 2,0000 |
|  | 50 | 3,0000 | 3,0000 | 3,0000 | 3,0000 | 3,0000 | 3,0000 | 3,0000 | 3,0000 | 3,0000 | 2,0000 |
|  | 75 | 3,0000 | 3,0000 | 3,0000 | 3,0000 | 3,0000 | 3,0000 | 3,0000 | 3,0000 | 3,0000 | 3,0000 |

| **Statistics Usability - Sphenoid Sinus** | | | | | | | | | | | |
| --- | --- | --- | --- | --- | --- | --- | --- | --- | --- | --- | --- |
|  | | SS Intraoperative Camerahandling | SS Ergonomics | SS Weight of Endoscope | SS Nausea | SS Dizziness | SS Headache | SS Positioning of Endoscope | SS Time for Preops | SS Conflict with Instruments | SS Lens cleaning Effort |
| N | Valid | 60 | 60 | 60 | 60 | 60 | 60 | 60 | 60 | 60 | 60 |
|  | Invalid | 20 | 20 | 20 | 20 | 20 | 20 | 20 | 20 | 20 | 20 |
| Mean | | 2,8500 | 2,9667 | 3,0833 | 2,9833 | 2,9333 | 2,9833 | 2,7667 | 2,6500 | 2,9500 | 2,1000 |
| Standarderror of the Mean | | ,06210 | ,04734 | ,04312 | ,01667 | ,03247 | ,01667 | ,05997 | ,06210 | ,02837 | ,07011 |
| Median | | 3,0000 | 3,0000 | 3,0000 | 3,0000 | 3,0000 | 3,0000 | 3,0000 | 3,0000 | 3,0000 | 2,0000 |
| Std.-Deviation | | ,48099 | ,36669 | ,33404 | ,12910 | ,25155 | ,12910 | ,46456 | ,48099 | ,21978 | ,54306 |
| Minimum | | 2,00 | 2,00 | 2,00 | 2,00 | 2,00 | 2,00 | 1,00 | 2,00 | 2,00 | 1,00 |
| Maximum | | 4,00 | 4,00 | 4,00 | 3,00 | 3,00 | 3,00 | 3,00 | 3,00 | 3,00 | 4,00 |
| Sum | | 171,00 | 178,00 | 185,00 | 179,00 | 176,00 | 179,00 | 166,00 | 159,00 | 177,00 | 126,00 |
| Percentile | 25 | 3,0000 | 3,0000 | 3,0000 | 3,0000 | 3,0000 | 3,0000 | 3,0000 | 2,0000 | 3,0000 | 2,0000 |
|  | 50 | 3,0000 | 3,0000 | 3,0000 | 3,0000 | 3,0000 | 3,0000 | 3,0000 | 3,0000 | 3,0000 | 2,0000 |
|  | 75 | 3,0000 | 3,0000 | 3,0000 | 3,0000 | 3,0000 | 3,0000 | 3,0000 | 3,0000 | 3,0000 | 2,0000 |

| **Statistics Usability - Frontal Sinus** | | | | | | | | | | | |
| --- | --- | --- | --- | --- | --- | --- | --- | --- | --- | --- | --- |
|  | | FS Intraoperative Camerahandling | FS Ergonomics | FS Weight of Endoscope | FS Nausea | FS Dizziness | FS Headache | FS Positioning of Endoscope | FS Time for Preops | FS Conflict with Instruments | FS Lens cleaning Efforts |
| N | Valid | 61 | 61 | 61 | 61 | 61 | 61 | 61 | 61 | 61 | 61 |
|  | Invalid | 19 | 19 | 19 | 19 | 19 | 19 | 19 | 19 | 19 | 19 |
| Mean | | 2,8033 | 2,9344 | 3,0984 | 2,9508 | 2,9344 | 2,9344 | 2,6721 | 2,6885 | 2,8689 | 2,2131 |
| Standarderror of the Mean | | ,06105 | ,05663 | ,04499 | ,02792 | ,03959 | ,03959 | ,07288 | ,05979 | ,04358 | ,08466 |
| Median | | 3,0000 | 3,0000 | 3,0000 | 3,0000 | 3,0000 | 3,0000 | 3,0000 | 3,0000 | 3,0000 | 2,0000 |
| Std.-Deviation | | ,47678 | ,44230 | ,35142 | ,21804 | ,30924 | ,30924 | ,56925 | ,46694 | ,34036 | ,66118 |
| Minimum | | 2,00 | 2,00 | 2,00 | 2,00 | 1,00 | 1,00 | 1,00 | 2,00 | 2,00 | 1,00 |
| Maximum | | 4,00 | 4,00 | 4,00 | 3,00 | 3,00 | 3,00 | 3,00 | 3,00 | 3,00 | 4,00 |
| Sum | | 171,00 | 179,00 | 189,00 | 180,00 | 179,00 | 179,00 | 163,00 | 164,00 | 175,00 | 135,00 |
| Percentile | 25 | 3,0000 | 3,0000 | 3,0000 | 3,0000 | 3,0000 | 3,0000 | 2,0000 | 2,0000 | 3,0000 | 2,0000 |
|  | 50 | 3,0000 | 3,0000 | 3,0000 | 3,0000 | 3,0000 | 3,0000 | 3,0000 | 3,0000 | 3,0000 | 2,0000 |
|  | 75 | 3,0000 | 3,0000 | 3,0000 | 3,0000 | 3,0000 | 3,0000 | 3,0000 | 3,0000 | 3,0000 | 2,0000 |
