# Supplementary material for: 3D endoscopy shows enhanced anatomical details and depth perception vs 2D: a multicentre study
Source: Eur Arch Otorhinolaryngol. 2020 Dec 29;278(7):2321–6. doi: 10.1007/s00405-020-06495-6 (PMC8165070; doi:10.1007/s00405-020-06495-6)
Supplement: Supplementary file 2 — Supplementary file2 (DOCX 29 KB) [file 405_2020_6495_MOESM2_ESM.docx]

| **Statistics Imaging - Ethmoid Sinus** | | | | | | | | | |
| --- | --- | --- | --- | --- | --- | --- | --- | --- | --- |
|  | | ES Recognition of Details | ES Color Brilliance | ES Illumination | ES Image Distortion | ES Size of Field | ES Depth Perception | ES Fogging | ES 3D Effect |
| N | Valid | 78 | 78 | 78 | 78 | 78 | 78 | 78 | 78 |
|  | Invalid | 2 | 2 | 2 | 2 | 2 | 2 | 2 | 2 |
| Mean | | 3,2692 | 2,7308 | 2,7564 | 2,8462 | 2,8333 | 3,8846 | 2,3333 | 4,2179 |
| Standarderror of the Mean | | ,11638 | ,09064 | ,07782 | ,05186 | ,05294 | ,08157 | ,06746 | ,09767 |
| Median | | 3,0000 | 3,0000 | 3,0000 | 3,0000 | 3,0000 | 4,0000 | 2,0000 | 4,0000 |
| Std.-Deviation | | 1,02783 | ,80054 | ,68729 | ,45803 | ,46756 | ,72040 | ,59580 | ,86261 |
| Minimum | | 1,00 | 2,00 | 1,00 | 1,00 | 2,00 | 2,00 | 1,00 | 1,00 |
| Maximum | | 5,00 | 5,00 | 4,00 | 4,00 | 4,00 | 5,00 | 4,00 | 5,00 |
| Sum | | 255,00 | 213,00 | 215,00 | 222,00 | 221,00 | 303,00 | 182,00 | 329,00 |
| Percentile | 25 | 3,0000 | 2,0000 | 2,0000 | 3,0000 | 3,0000 | 3,0000 | 2,0000 | 4,0000 |
|  | 50 | 3,0000 | 3,0000 | 3,0000 | 3,0000 | 3,0000 | 4,0000 | 2,0000 | 4,0000 |
|  | 75 | 4,0000 | 3,0000 | 3,0000 | 3,0000 | 3,0000 | 4,0000 | 3,0000 | 5,0000 |

| **Statistics Imaging - Maxillary Sinus** | | | | | | | | | |
| --- | --- | --- | --- | --- | --- | --- | --- | --- | --- |
|  | | MS Recognition of Details | MS Color Brilliance | MS Illumination | MS Image Distortion | MS Size of Field | MS Depth Perception | MS Fogging | MS 3D Effect |
| N | Valid | 78 | 78 | 78 | 78 | 78 | 78 | 78 | 78 |
|  | Invalid | 2 | 2 | 2 | 2 | 2 | 2 | 2 | 2 |
| Mean | | 2,6282 | 2,5641 | 2,5513 | 2,8590 | 2,8077 | 3,6538 | 2,2949 | 3,8590 |
| Standarderror of the Mean | | ,08384 | ,07868 | ,07218 | ,04732 | ,05180 | ,08908 | ,05802 | ,11928 |
| Median | | 3,0000 | 2,0000 | 3,0000 | 3,0000 | 3,0000 | 4,0000 | 2,0000 | 4,0000 |
| Std.-Deviation | | ,74046 | ,69487 | ,63752 | ,41792 | ,45748 | ,78669 | ,51242 | 1,05343 |
| Minimum | | 1,00 | 2,00 | 1,00 | 2,00 | 2,00 | 2,00 | 1,00 | 1,00 |
| Maximum | | 4,00 | 4,00 | 4,00 | 4,00 | 4,00 | 5,00 | 4,00 | 5,00 |
| Sum | | 205,00 | 200,00 | 199,00 | 223,00 | 219,00 | 285,00 | 179,00 | 301,00 |
| Percentile | 25 | 2,0000 | 2,0000 | 2,0000 | 3,0000 | 3,0000 | 3,0000 | 2,0000 | 3,0000 |
|  | 50 | 3,0000 | 2,0000 | 3,0000 | 3,0000 | 3,0000 | 4,0000 | 2,0000 | 4,0000 |
|  | 75 | 3,0000 | 3,0000 | 3,0000 | 3,0000 | 3,0000 | 4,0000 | 3,0000 | 5,0000 |

| **Statistics Imaging - Sphenoid Sinus** | | | | | | | | | |
| --- | --- | --- | --- | --- | --- | --- | --- | --- | --- |
|  | | SS Recognition of Details | SS Color Brilliance | SS Illumination | SS Image Distortion | SS Size of Field | SS Depth Perception | SS Fogging | SS 3D Effect |
| N | Valid | 60 | 60 | 60 | 60 | 60 | 60 | 60 | 60 |
|  | Invalid | 20 | 20 | 20 | 20 | 20 | 20 | 20 | 20 |
| Mean | | 3,1667 | 2,6000 | 2,6667 | 2,9333 | 2,8500 | 4,1167 | 2,3000 | 4,3500 |
| Standarderror of the Mean | | ,11924 | ,09267 | ,09408 | ,04674 | ,05737 | ,07551 | ,06848 | ,10313 |
| Median | | 3,0000 | 2,0000 | 3,0000 | 3,0000 | 3,0000 | 4,0000 | 2,0000 | 4,5000 |
| Std.-Deviation | | ,92364 | ,71781 | ,72875 | ,36204 | ,44436 | ,58488 | ,53043 | ,79883 |
| Minimum | | 1,00 | 2,00 | 1,00 | 2,00 | 2,00 | 3,00 | 1,00 | 1,00 |
| Maximum | | 5,00 | 5,00 | 5,00 | 4,00 | 4,00 | 5,00 | 4,00 | 5,00 |
| Sum | | 190,00 | 156,00 | 160,00 | 176,00 | 171,00 | 247,00 | 138,00 | 261,00 |
| Percentile | 25 | 3,0000 | 2,0000 | 2,0000 | 3,0000 | 3,0000 | 4,0000 | 2,0000 | 4,0000 |
|  | 50 | 3,0000 | 2,0000 | 3,0000 | 3,0000 | 3,0000 | 4,0000 | 2,0000 | 4,5000 |
|  | 75 | 4,0000 | 3,0000 | 3,0000 | 3,0000 | 3,0000 | 4,0000 | 3,0000 | 5,0000 |

| **Statistics Imaging - Frontal Sinus** | | | | | | | | | |
| --- | --- | --- | --- | --- | --- | --- | --- | --- | --- |
|  | | FS Recognition of Details | FS Color Brilliance | FS Illumination | FS Image Distortion | FS Size of Field | FS Depth Perception | FS Fogging | FS 3D Effect |
| N | Valid | 61 | 61 | 61 | 61 | 61 | 61 | 61 | 61 |
|  | Invalid | 19 | 19 | 19 | 19 | 19 | 19 | 19 | 19 |
| Mean | | 3,1148 | 2,6066 | 2,5410 | 2,8361 | 2,8689 | 3,9672 | 2,3115 | 4,2131 |
| Standarderror of the Mean | | ,11475 | ,09137 | ,09225 | ,06264 | ,05949 | ,09044 | ,07590 | ,12613 |
| Median | | 3,0000 | 3,0000 | 2,0000 | 3,0000 | 3,0000 | 4,0000 | 2,0000 | 4,0000 |
| Std.-Deviation | | ,89626 | ,71365 | ,72050 | ,48923 | ,46459 | ,70633 | ,59276 | ,98514 |
| Minimum | | 1,00 | 1,00 | 1,00 | 1,00 | 2,00 | 2,00 | 1,00 | 1,00 |
| Maximum | | 4,00 | 4,00 | 4,00 | 4,00 | 4,00 | 5,00 | 4,00 | 5,00 |
| Sum | | 190,00 | 159,00 | 155,00 | 173,00 | 175,00 | 242,00 | 141,00 | 257,00 |
| Percentile | 25 | 2,5000 | 2,0000 | 2,0000 | 3,0000 | 3,0000 | 4,0000 | 2,0000 | 4,0000 |
|  | 50 | 3,0000 | 3,0000 | 2,0000 | 3,0000 | 3,0000 | 4,0000 | 2,0000 | 4,0000 |
|  | 75 | 4,0000 | 3,0000 | 3,0000 | 3,0000 | 3,0000 | 4,0000 | 3,0000 | 5,0000 |
